# A fast and intuitive method for calculating dynamic network reconfiguration and node flexibility

**DOI:** 10.3389/fnins.2023.1025428

**Published:** 2023-02-09

**Authors:** Narges Chinichian, Johann D. Kruschwitz, Pablo Reinhardt, Maximilian Palm, Sarah A. Wellan, Susanne Erk, Andreas Heinz, Henrik Walter, Ilya M. Veer

**Affiliations:** ^1^Institute for Theoretical Physics, Technical University of Berlin, Berlin, Germany; ^2^Department of Psychiatry and Psychotherapy, Charité Campus Mitte (CCM), Charité-Universitätsmedizin Berlin, Freie Universität Berlin and Humboldt-Universität zu Berlin, Berlin, Germany; ^3^Bernstein Center for Computational Neuroscience, Berlin, Germany; ^4^Research Centre (SFB 940) “Volition and Cognitive Control”, Technische Universität Dresden, Dresden, Germany; ^5^Department of Philosophy and Humanities, Freie Universität Berlin, Berlin, Germany; ^6^Department of Mathematics and Computer Science, Freie Universität Berlin, Berlin, Germany; ^7^Faculty of Philosophy, Berlin School of Mind and Brain, Humboldt-Universität zu Berlin, Berlin, Germany; ^8^Department of Developmental Psychology, University of Amsterdam, Amsterdam, Netherlands

**Keywords:** task-based fMRI, dynamic functional connectivity, network neuroscience, template-based flexibility, community detection, dynamical network analysis, modular structure

## Abstract

Dynamic interactions between brain regions, either during rest or performance of cognitive tasks, have been studied extensively using a wide variance of methods. Although some of these methods allow elegant mathematical interpretations of the data, they can easily become computationally expensive or difficult to interpret and compare between subjects or groups. Here, we propose an intuitive and computationally efficient method to measure dynamic reconfiguration of brain regions, also termed flexibility. Our flexibility measure is defined in relation to an a-priori set of biologically plausible brain modules (or networks) and does not rely on a stochastic data-driven module estimation, which, in turn, minimizes computational burden. The change of affiliation of brain regions over time with respect to these a-priori template modules is used as an indicator of brain network flexibility. We demonstrate that our proposed method yields highly similar patterns of whole-brain network reconfiguration (i.e., flexibility) during a working memory task as compared to a previous study that uses a data-driven, but computationally more expensive method. This result illustrates that the use of a fixed modular framework allows for valid, yet more efficient estimation of whole-brain flexibility, while the method additionally supports more fine-grained (e.g. node and group of nodes scale) flexibility analyses restricted to biologically plausible brain networks.

## 1. Introduction

Over the past decades, a paradigm shift has taken place in studying the human brain, moving from a local to a more network-based perspective, giving rise to the field of network neuroscience. This evolution has, in part, been driven by the concept of graphs in math. A graph (network) consists of a set of vertices (nodes), which are connected by edges (links). In neuroimaging-based network neuroscience, brain regions identified by any given method of parcellation are considered the nodes of the network, while links can either be defined as white matter connections between brain regions (structural networks) or as statistical interdependencies between the time series of brain regions (functional networks) (Bondy and Murty, 2008; Fair et al., 2009; Power et al., 2010; Rubinov and Sporns, 2010; Sporns, 2010, 2012; Fornito et al., 2016).

Mesoscopic structures or groups formed by interactions between nodes of a network, called modules, clusters or communities, can be quantified by a variety of detection methods (Fortunato, 2010). Nodal interactions are typically represented by an adjacency matrix (*A*) of the network, where each element *i, j* of *A* (called *a*_*ij*_) is the weight of the connection or strength of interaction between nodes i and j. Modules are usually determined based on the general idea of maximizing the number/weight of within-group and minimizing the number/weight of between-group links. Modules can then be considered as entities in the network that can be modified individually without affecting the rest of the network. Modularity measures have been shown to be useful as a biomarker of disease, including epilepsy (Chavez et al., 2010), Alzheimer's disease (Brier et al., 2014), schizophrenia, bipolar, and major depressive disorder (Ma et al., 2020). However, brain modularity has also been associated with normal variation in cognition: Individuals with lower whole-brain modularity performed better in complex tasks, while those with higher modularity showed an advantage in simple tasks (Yue et al., 2017). Whereas the ‘static' community detection methods employed in the above-mentioned studies consider the brain's connectivity averaged over time (based on only one adjacency matrix per subject as a single-layer network), other methods have assessed changes in community structure over time (Meunier et al., 2009; Newell et al., 2009; Bassett et al., 2011; Calhoun et al., 2014; Alavash et al., 2015; Sporns and Betzel, 2016). These dynamic approaches take into account that a node can frequently change its connections depending on which state the brain is in, both during resting-state (RS) and during the performance of tasks. Here, changes in modular structure are captured by a sequence of adjacency matrices (*A*_*t*_), thus creating multi-layer networks. The adjacency matrices are typically calculated using a sliding-window approach on nodal time series, in which the window length reflects the time scale of interest (Fornito et al., 2016). Subsequently, dynamic module detection methods can be applied to these time-dependent multi-layer networks to not only characterize changes of modules over time, but also to determine how nodes change their affiliation [the module/group they belong to] as a function of time. The latter can be thought of as the flexibility of a node (Bassett et al., 2011; Betzel and Bassett, 2017) and is defined based on the consecutive presence of nodes in different modules over time (Meunier et al., 2010; Calhoun et al., 2014). These measures of flexibility enable us to track time-dependent changes and thereby track phenomena of both integration and segregation in the brain (Bassett et al., 2011; Braun et al., 2015). It offers the opportunity to study which brain nodes are more likely to change their affiliation over time and thereby which brain regions are rather consistently associated with a certain brain module, forming a backbone for the constantly changing network. For example, a recent study by Harlalka et al. (2019) suggested higher symptom severity in autism spectrum disorder to be associated with more connectivity flexibility in visual and sensorimotor areas during rest. Braun et al. (2015) demonstrated that individuals with more connectivity flexibility in frontal cortices have enhanced memory performance and score better on neuropsychological tests measuring cognitive flexibility, suggesting that dynamic network reconfiguration may form a fundamental mechanism underlying executive function. For a broader discussion on modularity and flexibility findings, see Karwowski et al. (2019).

A data driven widely used method to calculate brain network flexibility is based on the Louvain community detection algorithm by Blondel et al. (2008). This algorithm aims to optimize the variable *Q*, initially introduced for a single layer network by Newman (2006), and later modified for multi-layer networks by others (Mucha et al., 2010; Bazzi et al., 2016; Vaiana and Muldoon, 2018).


(1) $$\begin{array}{l} Q=\frac{1}{\mu }\underset{ijsr}{\sum }\left[\left(A_{ijs}-\gamma_{s}\frac{k_{is}k_{js}}{2m_{s}}\right)\delta_{sr}+\delta_{ij}C_{jsr}\right]\delta \left(c_{is},c_{jr}\right) \end{array}$$

More specifically: Where *A* is the Adjacency matrix of the network, *A*_*ijs*_ is the weight of connection between nodes *i* and *j* in layer *s*. γ_*s*_ is the resolution parameter for layer *s*, *i* and *j* are indices of nodes, and *s* and *r* indices of layers. *k*_*is*_ is the degree of node *i* in layer *s*. *m*_*s*_ is proportional to the sum of weights in layer *s*. *C*_*jsr*_ refers to the connection of node *j* to itself in different layers. *c*_*is*_ is the defined module/cluster of node *i* in layer *s*. Finally, *Q* captures how good the grouping is compared to a null-model (here random).

Although, this and similar methods have undoubtedly contributed to our understanding of brain dynamics, these come with a cost: Given the random nature of algorithms like Louvain, the resulting clusters may differ each time the algorithm is run on the same adjacency matrix. As such, brain modules show variation within and across participants, which is overcome by running the algorithm multiple times to reach a consensus on the modular structure (Lancichinetti and Fortunato, 2012). However, this can be a computationally expensive process, while the identified modules may in the end have low biological plausibility or at least cannot be interpreted straightforwardly.

Here, we introduce a new method to capture nodal flexibility and brain network reconfiguration using a fast and intuitive method based on a set of template modules. This offers three main advantages over the existing methods:

1. It is computationally more efficient and deterministic compared to the Louvain (and similar) algorithm.
2. It offers high replicability, as it uses the same set of module templates for all subjects and time scales. This ensures comparability between subjects and studies, which is one of the current concerns in the field (Hallquist and Hillary, 2018).
3. It gives researchers the opportunity to choose the best-fitting, or biologically most relevant module templates for each study.

Although the exact computational complexity of the Louvain algorithm is not mentioned in the literature, it is suggested to be essentially linear in the number of links in the graph (Lancichinetti and Fortunato, 2009).^1^ But the complexity mentioned is regarding the one time run of the greedy algorithm. The Louvain algorithm starts with assigning a distinct community to each network node. In the initial phase then, there are as many communities as nodes. It then evaluates the gain in modularity [difference between Q values for different cases] that would result from removing each node i from its community and placing it in the community of j for each of its neighbors j. The i-th node is then placed in the community with the greatest positive gain. If there is no positive gain, node i remains in its original community. This process is repeated until no further improvement is possible, at which point the first phase is finished. This first phase concludes when a local modularity maximum is reached and no individual move can improve modularity. The output of the algorithm is dependent on the order in which the nodes are considered. The second phase of the algorithm involves the construction of a new network whose nodes are the communities discovered in the first phase. To accomplish this, the weights of the links between the new nodes are determined by adding the weights of the links between nodes in the respective two communities. In the new network, links between nodes of the same community result in self-loops for this community. Once this second phase is complete, the algorithm's initial phase can be reapplied to the resulting weighted network again. This 2-phase process is repeated until there are no more modifications and maximum modularity is achieved. A partitioning of the network is achieved through this process of repeating the 2-phase until the Q cannot be improved, but to find a reliable final representative partition that doesn't depend on the order in which the algorithm chooses the nodes, this whole process is repeated several times until a consensus is reached (Blondel et al., 2008). On the other hand, our template-based method, gives the same deterministic value each time and does not need repetition or a consensus-finding step. We believe that the deterministic nature of the template-method can be interpreted as the intrinsic “efficiency factor”. The sum is always linear to the number of links and we need one time of adding the weights to find the total weight of connections to each module.

In this work we describe our proposed method in detail and apply it to a real-life dataset that was previously assessed using a Louvain-like locally greedy heuristic algorithm (Blondel et al., 2008; Braun et al., 2015). Compared to the previous work, we demonstrate that our method is equally successful in capturing a brain reconfiguration pattern that mimics the stimulation periods of an externally-cued working memory task, yet in our case can be directly related to well-known functional brain networks as well.

## 2. Methods

### 2.1. Concept and steps

**Figure 1:**
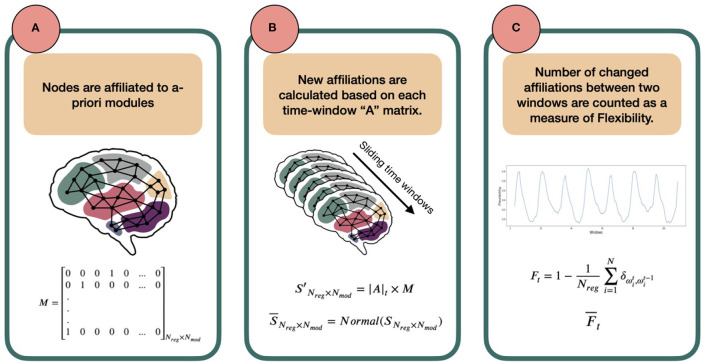
Schematic overview of the template-based flexibility method. **(A)** Each node has an a-priori affiliation to a template module, not allowing overlap. In this paper, we use the Brainnetome atlas for node definition (Fan et al., 2016) and the FIND Lab network templates as predefined modules [http://findlab.stanford.edu/; Shirer et al., 2012]. Importantly, matrix M, describing the a-priori module affiliation for each node, is predetermined and serves as a reference. **(B)** Using a sliding-window approach, an adjacency matrix is constructed for each time window by calculating Pearson correlation coefficients between the time series of all possible pairs of nodes. Then, for each node and time window the reference module receiving the highest normalized connection weight will serve as the new modular affiliation for that node in that time window. **(C)** Last, the number of affiliation changes between affiliation vector in *t* and its successive vector in *t* + 1 is defined as the flexibility *F*_*t*_ of the network between two time points. The average of *F*_*t*_ across participants (called $\bar{F_{t}}$) can be plotted for all consecutive time points (an example presented later in Figure 3).

Before going into mathematical detail, let us first explain the concept behind the method. Consider the brain as a network, in which each region of the brain (defined by any arbitrary parcellation) is a node, each co-activation between any two nodes is an edge, and each node belongs to an a-priori defined set of nodes, termed a module. As a first step, we consider that each node has an a-priori affiliation to one of the predefined template modules or in other words, belongs to an a-prioiri template module. The affiliation is determined as the template module with which each node has the largest spatial overlap. Next, the strengths of all edges between each node and all members of every module are summed. When a node is more strongly connected to nodes affiliated with another module than to nodes of its own predefined module, then this node will receive another affiliation than its a-priori one. This can now be extended to a dynamic scenario, in which node affiliations can be determined for a range of consecutive time points. Some nodes might change their affiliation over time, while others do not. The ratio of nodes changing affiliation with respect to all nodes is what we are interested in. We understand this ratio as a measure of flexibility of the brain. In other words, the more nodes switch affiliation between consecutive time points, the more flexibility in network dynamics we assume. See Figure 1 for a summary of these steps.

The steps to calculate this flexibility measure are listed below in detail:

1. An a-priori affiliation is assigned to each node to form the following matrix M:
  (2) $$\begin{array}{l} M={\left[\begin{array}{ccccccc} 0 & 0 & 0 & 1 & 0 & \ldots & 0\\ 0 & 1 & 0 & 0 & 0 & \ldots & 0\\ .\\ .\\ .\\ 1 & 0 & 0 & 0 & 0 & \ldots & 0 \end{array}\right]}_{N_{reg}\times N_{mod}} \end{array}$$
  Where *N*_*reg*_ is the number of regions (nodes) and *N*_*mod*_ number of a-priori modules. Each row of this matrix belongs to a node and, in the first-approximation case in this paper, has only one non-zero element that indicates the a-priori modular affiliation of the node. For example, in row 1 the fourth column is 1, which means that the first node has an a-priori affiliation to template module 4.
  Note that we assign all nodes that do not show any overlap with the template modules to a last, artificial module to not exclude these nodes in calculating the flexibility metric.
2. Next, for each node we extract the mean time series across all volumes of the fMRI scan. We then divide our time-series into smaller windows using a sliding-window approach. For each time window, an adjacency matrix is constructed using Pearson correlation coefficients between all possible node pairs. The adjacency matrix at time-window *t* is defined as *A*_*t*_ of shape *N*_*reg*_ × *N*_*reg*_:
  (3) $$\begin{array}{l} A_{t}=\left[\begin{array}{cccc} Weighted & Adjacency & Matrix\\ of & Time & Window & t \end{array}\right] \end{array}$$
3. Now, we want to calculate how each node is connected to the nodes that are the predefined members of each of the template modules, as defined in *M*. To this end, we sum the absolute values of all the weights from one node to all the nodes affiliated to each of the modules, so that each node has *N*_*mod*_ [in our subsection 2.2 analysis: 15] different values (one weighted sum for links to each module), indicating the strength of its links with the predefined members of each of the template modules. In mathematical terms, we calculate the matrix *S*′ as follows:
  (4) $$\begin{array}{l} S{{\;}^{\prime }}_{N_{reg}\times N_{mod}}\;=\;|A{|}_{t}\times M \end{array}$$
  where |*A*|_*t*_ matrix elements are the absolute values of *A*_*t*_ elements and the matrix has the dimension *N*_*reg*_ × *N*_*reg*_. Row i of *S*′ belongs to the region *i* and each column *j* shows the sum of absolute connection weights of *i* to the members of *j*-th module. As the predefined modules differ in size, the *S*′ matrix elements are then normalized to the number of regions affiliated by template definition to the modules, creating a new matrix called *S* [dividing each matrix element $S_{ij}^{\prime }$ by the number of regions affiliated to the *j*th template module]. Importantly, to be able to compare the elements of *S*, we normalize it in a way that the sum of each row is one. This normalization step has no effect on the output of the next steps but is rather to increase the interpretability at this stage. The normalized numbers thus represent which portion of each node's connections is to which module. We call this new matrix, $\bar{S}$.
  (5) $$\begin{array}{l} {\bar{S}}_{N_{reg}\times N_{mod}}=Normal\left(S_{N_{reg}\times N_{mod}}\right) \end{array}$$
4. With $\bar{S}$, we have the ratio of affiliations to each module calculated for all nodes. From these, the strongest module affiliation per node is chosen as the winner which together form an affiliation vector for time window t; we call this vector Ω_*t*_:
  (6) $$\begin{array}{l} \Omega_{t}={\left[\begin{array}{c} ArgMax\left({\bar{S}}_{1*}\right)\\ ArgMax\left({\bar{S}}_{2*}\right)\\ ArgMax\left({\bar{S}}_{3*}\right)\\ ArgMax\left({\bar{S}}_{4*}\right)\\ ..\\ ArgMax\left({\bar{S}}_{i*}\right)\\ \ldots \\ ArgMax\left({\bar{S}}_{N_{reg}*}\right) \end{array}\right]}_{N_{reg}\times 1} \end{array}$$
  where $ArgMax\left({\bar{S}}_{i*}\right)$ points to the name/number (argument) of the winner module in row i of matrix $\bar{S}$.
5. Following steps 2-4 for consecutive time windows, we calculate one Ω_*t*_ for each window t. The *flexibility* of the network denoted by *F* is then defined as the ratio of regions that change their affiliation from one window to the next to the total number of network regions, or:
  (7) $$\begin{array}{l} F_{t}=1-\frac{1}{N_{reg}}\overset{N}{\underset{i=1}{\sum }}\delta_{\omega_{i}^{t},\omega_{i}^{t-1}}, \end{array}$$
  where ω denotes an element of vector Ω. The Kronecker delta $\delta_{\omega_{i}^{s},\omega_{j}^{t}}$ is 1 if $\omega_{i}^{s}=\omega_{j}^{t}$ and 0 otherwise. The ∑ then counts the number of nodes that did not change their affiliation between windows *t* and *t* + 1. Note that as a side-product of calculating Ω, we can output a vector describing the affiliations over time for each node separately as well by making a vector of the same element in Ω_*t* = 1, ..,_*N*__*t*__:
  (8) $$\begin{array}{l} {\left[\begin{array}{c} \omega_{i}^{t=1},\omega_{i}^{t=2},\omega_{i}^{t=3}\ldots ,\omega_{i}^{t=N_{t}} \end{array}\right]}_{1\times N_{t}} \end{array}$$
  Where *N*_*t*_ is the total number of time windows. This output can be used for further region-specific analysis.
6. Where we apply the method to real-life data (see subsection 2.2) we also calculate the average *flexibility* over time for a sample (cohort of subjects), ${\bar{F}}_{t}$, by simply summing the flexibility over all participants and divide it by the sample size (*N*_*sub*_).

### 2.2. Application on a previously studied dataset

**Figure 2:**
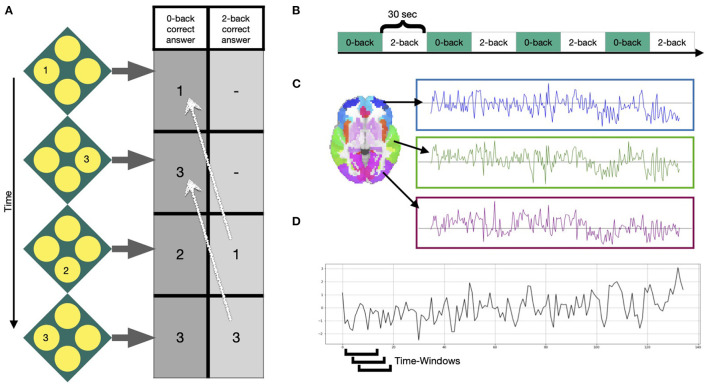
Task and signals. **(A)** Example of the N-back working memory task with a 0-back and 2-back condition, during which participants were asked to choose the value that was either shown at the current step or 2 steps ago, respectively. **(B)** Four blocks of each condition were presented in alternated fashion for 30 s. **(C)** After preprocessing, mean time courses were extracted from 246 Brainnetome atlas regions (Fan et al., 2016). **(D)** Windowed time series were extracted using a sliding-window approach, moving a window of 15 time points over the time series one volume at a time.

In our application study, we used 331 participants of the 344 participants included in Braun et al. (2015): Thirteen subjects were excluded due to scanning artifacts, exceeding movement or insufficient image quality. Functional MRI data were acquired at three sites during performance of an N-back task: the Life and Brain Center of the University of Bonn, the Central Institute of Mental Health Mannheim, and Charité - Universitätsmedizin Berlin. The study was approved by the Medical Ethics Committee of the three study sites and all participants provided written informed consent. At all sites, a Siemens Trio 3T MRI scanner (Siemens Healthcare, Erlangen, Germany) was used with identical sequences: gradient-echo EPI, 28 slices, slice thickness 4mm (1mm gap), field of view 192 x 192 x 140 mm, acquisition matrix 64 x 64, TR (repetition time) 2s, TE (echo time) 30 ms, flip angle 80°. The task was presented in a blocked fashion. Four blocks of 0-back and 2-back each (30s duration) were alternated, starting with the 0-back condition. Participants were asked to either press the button corresponding to the number shown on the screen (0-back) or the number that was shown 2 steps ago (2-back). See Figure 2 for more information on the task. Python packages nilearn, Scikit-learn and matplotlib are used for visualization purposes in this manuscript (Hunter, 2007; Pedregosa et al., 2011). Standard preprocessing was conducted using SPM8 (Penny et al., 2011) and included motion correction (participants with >3mm translation and >1.7° rotation between volumes were excluded), slice-time correction, spatial smoothing with a FWHM of 9 mm, high-pass temporal filtering with a 128s cutoff, and normalization to the Montreal Neurological Institute (MNI) template space with 3 mm isotropic voxel size. A detailed description of data acquisition and preprocessing is provided in Esslinger et al. (2009). Mean time-courses of the 246 Brainnetome Atlas regions (Fan et al., 2016) were extracted from the preprocessed data of the 331 subjects. In line with Braun et al. (2015), a 15-volume window length with 14 volumes overlap was chosen for the sliding-window analysis (Figures 2C, D), generating in total 114 windows for each subject. For every window, we calculated an adjacency matrix using Pearson correlation coefficients between all possible pairs of the 246 regions mean time series [using scipy.stats.pearsonr Virtanen et al. (2020)]. Considering that the N-back working memory task consisted of 30 s alternating blocks of 0-back and 2-back, the 15-volume window (30 s length) allows for one window purely reflecting a single condition block. For more information on selection of the window length see Braun et al. (2015) and Leonardi and Ville (2015).

The a-priori modules (Matrix M) were selected based on 14 well-described functional connectivity template networks (modules) in Shirer et al. (2012) by the FIND lab (http://findlab.stanford.edu/). As described before, a 15th (artificial) module was added comprising all atlas regions that did not overlap with any of the 14 template networks. The a-priori affiliations of all atlas regions can be found in Table 1 and the labels of the FIND lab templates in Table 2.

**Table 1:** Region a-priori Affiliation, columns marked “R” are region numbers and “M” columns are a-priori modular affiliations.

**R**	**M**	**R**	**M**	**R**	**M**	**R**	**M**	**R**	**M**	**R**	**M**	**R**	**M**	**R**	**M**	**R**	**M**	**R**	**M**
1	1	26	11	51	6	76	2	101	15	126	13	151	9	176	4	201	5	226	3
2	1	27	7	52	11	77	1	102	15	127	13	152	9	177	9	202	5	227	3
3	7	28	11	53	12	78	8	103	13	128	14	153	4	178	4	203	5	228	3
4	11	29	14	54	12	79	2	104	13	129	14	154	4	179	4	204	5	229	3
5	4	30	14	55	12	80	6	105	5	130	14	155	12	180	1	205	5	230	3
6	4	31	14	56	12	81	7	106	5	131	8	156	12	181	13	206	5	231	3
7	1	32	11	57	12	82	15	107	14	132	8	157	2	182	13	207	9	232	3
8	1	33	6	58	12	83	6	108	14	133	14	158	2	183	1	208	9	233	3
9	1	34	11	59	12	84	15	109	15	134	14	159	14	184	1	209	14	234	3
10	1	35	6	60	12	85	6	110	15	135	13	160	14	185	4	210	14	235	8
11	1	36	6	61	2	86	6	111	13	136	13	161	12	186	4	211	15	236	8
12	1	37	1	62	2	87	6	112	13	137	7	162	12	187	4	212	15	237	4
13	4	38	1	63	14	88	6	113	13	138	11	163	8	188	4	213	15	238	4
14	4	39	6	64	14	89	15	114	13	139	14	164	15	189	5	214	15	239	8
15	1	40	1	65	13	90	15	115	15	140	14	165	1	190	5	215	4	240	3
16	11	41	4	66	8	91	14	116	15	141	8	166	1	191	10	216	4	241	4
17	7	42	4	67	12	92	14	117	15	142	11	167	1	192	10	217	4	242	3
18	11	43	6	68	12	93	15	118	15	143	6	168	1	193	5	218	4	243	3
19	1	44	11	69	15	94	15	119	13	144	6	169	8	194	10	219	3	244	3
20	1	45	15	70	15	95	7	120	15	145	8	170	8	195	10	220	3	245	3
21	1	46	15	71	2	96	15	121	6	146	8	171	15	196	5	221	3	246	3
22	11	47	4	72	2	97	14	122	6	147	13	172	15	197	10	222	3		
23	7	48	4	73	2	98	14	123	6	148	13	173	1	198	10	223	8		
24	11	49	4	74	2	99	7	124	6	149	13	174	1	199	5	224	15		
25	14	50	15	75	6	100	15	125	13	150	13	175	4	200	5	225	3		

**Table 2:** Findlab-based modules (Shirer et al., 2012) used in our application section.

**Number**	**Name**
Module 1	Anterior Salience
Module 2	Auditory
Module 3	Basal Ganglia
Module 4	Dorsal Default Mode Network (dDMN)
Module 5	High Visual
Module 6	Language
Module 7	Left Executive Control (LECN)
Module 8	Posterior Salience
Module 9	Precuneus
Module 10	Prim Visual
Module 11	Right Executive Control (RECN)
Module 12	Sensorimotor
Module 13	Ventral Default Mode Network (vDMN)
Module 14	Task Positive
Module 15	Undefined

To obtain a broader view of the meso-scale dynamics, the modular allegiance matrix T and integration matrix R were calculated using the methods from Braun et al. (2015). Each element *t*_*i,j*_ of modular allegiance matrix T shows the ratio of windows where node i and j were present in the same module relative to all windows. To calculate the T for each condition, we separated windows with 80% of their time-points in one condition and ignored the others.

To calculate the integration matrix R with elements *r*_*k,l*_, which show the strength of co-working between modules k and l, when we have *N*_*mod*_ modules {*M*_1_, *M*_2_, …*M*_*N*_*mod*__}, we first use all the T matrix elements [link between two regions] with one end (region) in module k and the other end (region) in module l to extract I matrix elements (*i*_*k,l*_). It can be written as:


(9) $$\begin{array}{l} i_{k,l}=\frac{\sum_{i\in M_{k},j\in M_{l}}T_{i,j}}{\left|M_{k}||M_{l}\right|}, \end{array}$$

where k and l are two modules, |*M*_*k*_| shows the size of module *M*_*k*_. Then we normalize the I elements with division by internal connections of both modules and call the resulting elements elements of matrix R:


(10) $$\begin{array}{l} r_{k,l}=\frac{i_{k,l}}{\sqrt{i_{k,k}i_{l,l}}}, \end{array}$$

R is the integration matrix.

## 3. Results

**Figure 3:**
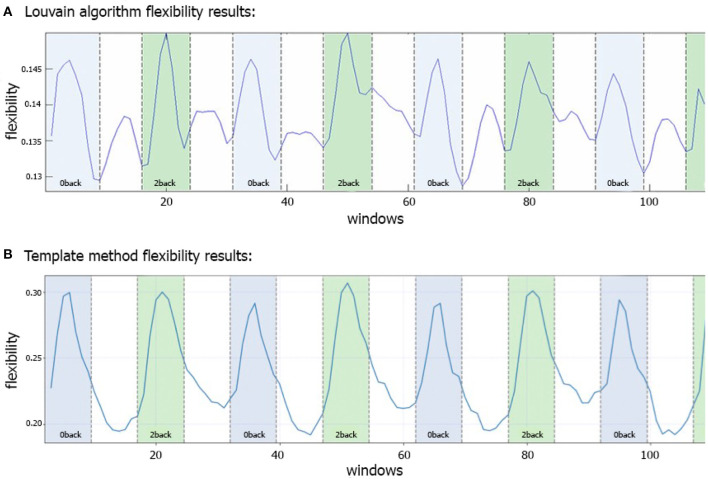
Comparison of flexibility generated by the generalized Louvain-like locally greedy heuristic algorithm (Blondel et al., 2008; Jeub et al., 2022) and the template-based method during an N-back working memory task. **(A)** Flexibility plot from Braun et al. (2015) illustrating the probability that a brain region changes its modular allegiance between two consecutive windows in a sample of 344 healthy subjects. The original plot is used with permission of the publisher. **(B)** Flexibility plot generated by the template-based method. Here, the flexibility number in each time-window is the fraction of regions that change their affiliation from one time window to the next (i.e., the number of changed regions divided by the total number of nodes). The plots are generated using a subset of 331 subjects from the same cohort as used in Braun et al. (2015). Note that in both plots a time window covers 15 EPI volumes with a TR of 2 s, corresponding to a window length of 30 s. The window was shifted with one volume at a time, allowing for 14 EPI volumes overlap between consecutive windows, which yielded 114 windows in total.

Figure 3A shows the N-back flexibility pattern across all nodes from Braun et al. (2015), while Figure 3B shows the pattern generated by our method when applied to the same dataset (331/344 subjects of the same sample). Similar to Figure 3A, the peaks illustrate maximum flexibility of the brain during performance of both the 0- and 2-back condition. In contrast, the transitions between the two task conditions coincide with troughs when applying our method, whereas Braun et al. (2015) described additional, yet smaller peaks during these transition phases when using the generalized Louvain algorithm. On average, higher flexibility is observed during the 2-back than 0-back blocks, although the difference is relatively small (*t* = −2.9, *p* = 0.03).

**Figure 4:**
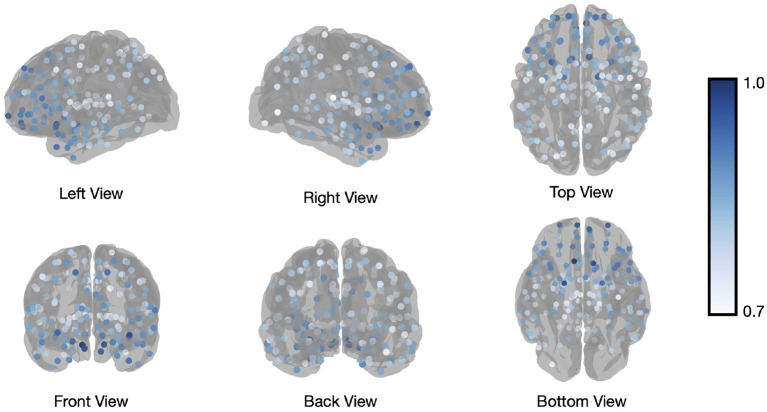
Brainnetome atlas brain regions switching. Number of affiliation switches between consecutive windows for regions of the Brainnetome Atlas, averaged across all subjects and normalized to the most frequently switching node to yield values between 0 and 1. The visualized regions are those with values higher than 0.7.

In addition to calculating flexibility across all nodes, we can use the information captured in the fifth step to describe the affiliation changes of each individual node. This allows us to have a closer look at which nodes switch their affiliation over time most frequently, or at how often the a-priori constituents of each of the template networks switch their affiliation. Figure 4 illustrates how many times each node (Brainnetome regions in our analysis) switches its affiliation between two consecutive windows. Note that the number of switches was normalized to the number of switches performed by the node that switched most frequently, forcing the latter node to have a value of 1 and the other nodes to have a value between 0 and 1. Nodes within the prefrontal cortex predominantly show affiliation changes over time during execution of the N-back task. This is in agreement with the previous findings (Owen et al., 2005; Cao et al., 2014; Braunlich et al., 2015; Minamoto et al., 2015).

**Figure 5:**
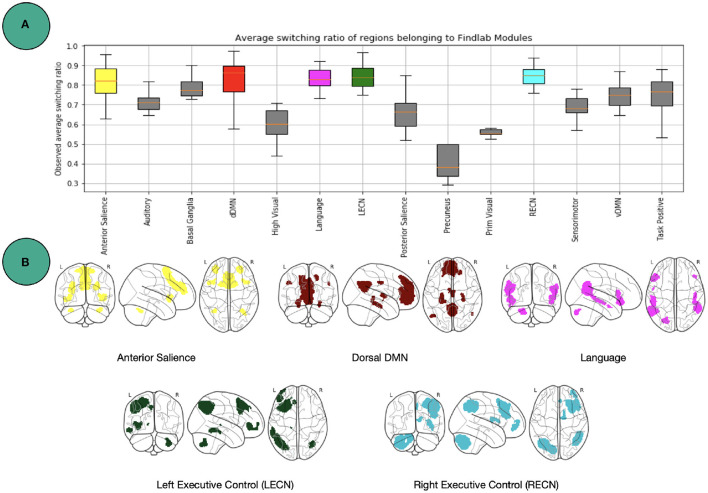
Findlab brain areas switching. **(A)** Average number of affiliation switches between consecutive windows for each FIND lab template network, averaged across all subjects. Abbreviations are listed in Table 2. **(B)** Illustration of the four template networks for which its constituent nodes demonstrated the highest flexibility [http://findlab.stanford.edu/; Shirer et al. (2012)]. See Figure 6 for more statistics.

**Figure 6:**
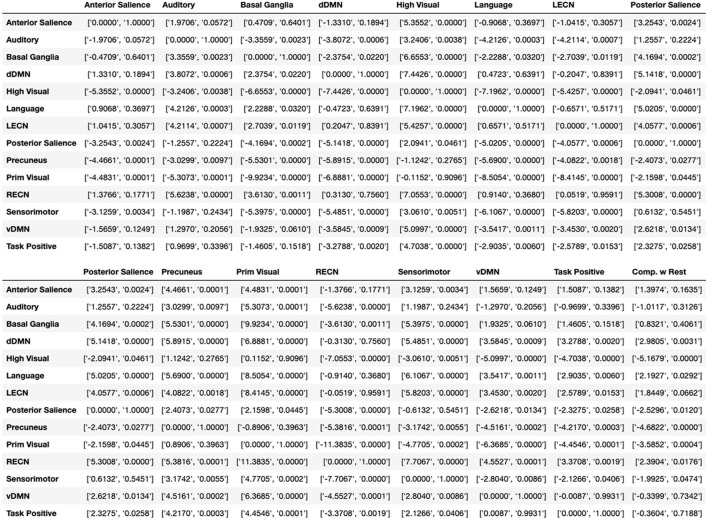
Additional statistics for Figure 5. Independent t and p values between boxplot modules in Figure 5, shown as [t-value, p-value]. The last column called “Comp. w Rest” calculates the t-test between the specific module and the whole brain. For visualization purpose the table is cut to two parts.

One level coarser at the module level, we can look at the average switching ratio of template modules. The boxplots in Figure 5 demonstrate for each of the FIND lab template modules how often their a-priori defined constituent nodes on average switch their modular affiliation over time across participants. Additional statistical analysis for modules in Figure 5 is provided in Figure 6. Constituent nodes of the default mode network (DMN), salience network (SN), left and right executive control network (L/RECN), and language network seemingly switch their affiliation most often during execution of the N-back task.

**Figure 7:**
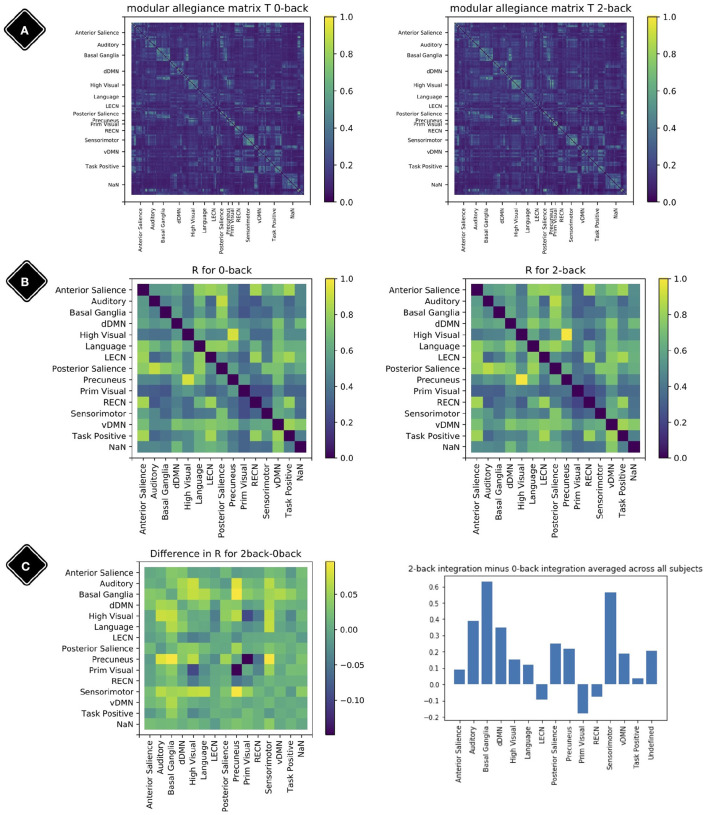
Modular allegiance and integration. Diagonal elements of the matrices are set to be zero. **(A)** Modular allegiance of the two conditions 2-back and 0-back; to calculate a T matrix for one condition, we used only the windows with 80% of their time-points in that condition. **(B)** Integration matrix for 0-back and 2-back. **(C)** Change in the integration values *R*_2−*back*_ − *R*_0−*back*_ (left plot) and sum of rows (from the left plot matrix) as each modules integration value (right plot).

Figure 7 shows the result of modular allegiance and integration analysis. We observe a general increase in integration values in 2-back compared to 0-back except for three modules. This overall increase in integration is in agreement with previous findings (Finc et al., 2020).

## 4. Discussion

**Figure 8:**
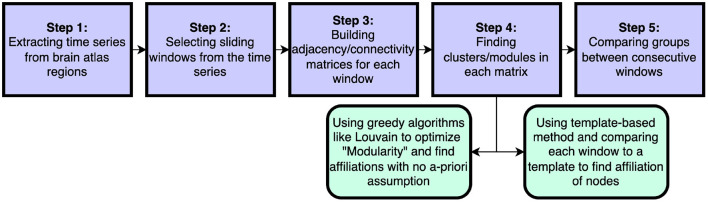
Schematic steps to calculate dynamical flexibility. The time series are extracted from brain scans. Selected sliding windows are used to generate adjacency/connectivity matrices. The groups/clusters/modules are found in each matrix^*^ using a feasible clustering method. In this step, the method of choice can be a well-known method like the optimization of Newman's modularity Q using greedy Louvain algorithm or it can be our template-based method that considers the a-priori information about brain as pre-assumption. Finally the assigned affiliations in windows are compared and the differences are found. ^*^In some methods, different sliding window matrices are put together to make a multi-layer network and then an adjusted version of modularity optimization is employed to find module through all layers.

In this work we introduce a new method to assess flexibility in analyses of dynamic functional connectivity. In the application section we set out to compare our method against the currently most used data-driven method described in Braun et al. (2015), in which the computationally more expensive generalized Louvain algorithm was applied to derive the modular structure of the data (Blondel et al., 2008; Mucha et al., 2010; Bassett et al., 2011; Jeub et al., 2022). See Figure 8 for a schematic comparsion of steps in standard vs. template flexibility calculations. We demonstrate that our method is able to reveal a flexibility pattern during the N-back working memory task that is highly similar to the pattern found in Braun et al. (2015). The most notable difference between the results obtained with our method and the Louvain algorithm was the absence of the small increase in flexibility during the transition of the 0- and 2-back blocks. Braun et al. (2015) interpret this to reflect “dual-task” performance. We suggest an alternative explanation based on the current results: increased flexibility may be needed for switching tasks at the start of each new condition block (shown as a delayed peak in the middle of the marked blocks), while less flexibility may be needed during prolonged execution of the task in each block (shown as a delayed trough exactly in between blocks). As such, the periods of lower flexibility may show the preferred brain configuration for the execution of the task blocks. A further more theoretical analysis of a simulated BOLD signal with block induced inputs might be helpful in interpreting the dual-task vs. no-dual-task hypothesis.

As has been shown abundantly in the literature, the prefrontal cortex plays an important role in the performance of working-memory tasks (Owen et al., 2005; Cao et al., 2014; Braunlich et al., 2015; Minamoto et al., 2015). Therefore, it is not surprising that we found nodes in the prefrontal cortex to show the most flexible behavior during execution of the N-back task. Moreover, at the modular level we see the highest flexibility in nodes that have an a-prori affiliation to the DMN, SN, L/RECN and language modules. The DMN is known to have an antagonistic relation with fronto-parietal networks, such as the L/RECN: when the latter is more active during cognitively demanding tasks (such as the N-back) the DMN is less active (Fox et al., 2005). Interestingly, a key role has been assigned to the SN in allocating neural resources between more internally (DMN) or externally (ECN) oriented processes (Uddin et al., 2011). Taken together, we see these results as further proof of our method's validity.

We discussed above how our method could be used to assess flexibility. That is, both on the network (module) level and at the regional (node) level, thereby extending the inferential potential compared to the other widely-used algorithms. However, our analytical procedure also offers possibilities for more fine-grained investigations of modular affiliations. In the description of our method and application analysis we determined the modular affiliation for a particular node and window as the module with which the node demonstrated the strongest connectivity in the affiliation vector. Although this is arguably the easiest and most pragmatic choice, it would also be possible to use the weighted affiliation with each of the template modules in the affiliation vector [Method section, step 3] to assess flexibility. Such a weighted approach may ultimately prove to be even more informative in characterizing brain flexibility. Another limitation of our method appears in the limits of a-priori module sizes. If the template modules are significantly different in size, a single division to the size of each module is not enough to account for the difference in the size of modules. In theory, one can define two a-priori modules of size 1 and *N*_*reg*_ − 1, but such a template definition would result in a flexibility which is very sensitive to the connection weights from that one single node. In spite of this, additional analysis revealed a comparable but not easily interpretable flexibility result if the nodes are randomly assigned to another similar-size a-priori module set and if, in a simplified case, the Pearson distance between different windows is used as a measure of flexibility [see chapter 6 of Chinichian (2022) for details]. This demonstrates that, in a larger context, the connectivity changes between successive windows can be tracked even before using a template. The researcher's choice of template provides an additional degree of freedom to find a suitable match for the research question. It allows researchers to focus on a subset of nodes that are relevant relative to the rest of the network, but it also introduces the limitation that results from different template selections may not be easily comparable. We recommend that every report on the template flexibility should include the exact template details [similar to Table 1] to allow for a fair interpretation of the results.

The current manuscript, focuses on the block-designed task fMRI which provides a fairly easy-to-interpret and comprehensible application case. A further investigating of resting-state fMRI and the changes in the flexibility during rest could provide more insight to the different aspects of this method. A study of resting-state fMRI from 95 subjects meeting criteria for Major Depressive Disorder and/or common anxiety disorders from the Netherlands Study of Depression and Anxiety (NESDA) is in preparation by a collaborator team (Dickhoff, 2022).

In conclusion, the method proposed in the current study is able to generate flexibility results that are highly comparable to the results obtained with a more sophisticated data-driven method. Besides having a much higher computational efficiency, our method also promotes replicability across different samples and studies through the use of biologically plausible template modules. We believe that our approach can be a feasible choice for researchers aiming to study dynamical reconfiguration at multiple scales of the brain, be it nodes, modules, or the brain as a whole.

## Data availability statement

The data analyzed in this study is subject to the following licenses/restrictions: Special permission is required from the project leaders. Participants' fMRI data is to be treated as confidential. Requests to access these datasets should be directed to Henrik.Walter@charite.de.

## Ethics statement

The studies involving human participants were reviewed and approved by Life and Brain Center of the University of Bonn, the Central Institute of Mental Health Mannheim, and Charité - Universitätsmedizin Berlin Medical Ethics Committee. The patients/participants provided their written informed consent to participate in this study.

## Author contributions

NC designed the method, the computational framework, analyzed the data, and wrote the manuscript. PR extracted the average time-series from the pre-processed data. IV and HW supervised the project. IV, HW, and JK contributed to research design and discussions of the manuscript. All authors discussed the results and commented on the manuscript.

## Footnotes

^1^Other implementations of the Newman-Girvan Q optimization are suggested to be of nlogn complexity (Lancichinetti and Fortunato, 2009; Blondel, 2022) in networks with a clear modular structure.

## References

[B1] Alavash M. Hilgetag C. C. Thiel C. M. Giessing C. (2015). Persistency and flexibility of complex brain networks underlie dual-task interference. Hum. Brain Mapp. 36, 3542–3562. 10.1002/hbm.22861 26095953 PMC6869626

[B2] Bassett D. S. Wymbs N. F. Porter M. A. Mucha P. J. Carlson J. M. Grafton S. T. (2011). Dynamic reconfiguration of human brain networks during learning. Proc. Nat. Acad. Sci. 108, 7641–7646. 10.1073/pnas.1018985108 21502525 PMC3088578

[B3] Bazzi M. Porter M. A. Williams S. McDonald M. Fenn D. J. Howison S. D. (2016). Community detection in temporal multilayer networks, with an application to correlation networks. Multiscale. Model Simul. 14, 1–41. 10.1137/15M1009615

[B4] Betzel R. F. Bassett D. S. (2017). Multi-scale brain networks. Neuroimage. 160, 73–83. 10.1016/j.neuroimage.2016.11.006 27845257 PMC5695236

[B5] Blondel V. (2022). The Louvain Method for Community Detection in Large Networks. Available online at: https://www.nasa.gov/nh/pluto-the-other-red-planet (accessed December 12, 2022).

[B6] Blondel V. D. Guillaume J.-L. Lambiotte R. Lefebvre E. (2008). Fast unfolding of communities in large networks. J. Stat. Mech. Theory Exp. 2008, 10008. 10.1088/1742-5468/2008/10/P1000821517554

[B7] Bondy J. A. Murty U. S. R. (2008). Graph Theory. New York: Springer. p. 244. 10.1007/978-1-84628-970-5

[B8] Braun U. Schafer A. Walter H. Erk S. Romanczuk-Seiferth N. Haddad L. . (2015). Dynamic reconfiguration of frontal brain networks during executive cognition in humans. Proc. Nat. Acad. Sci. 112, 11678–11683. 10.1073/pnas.1422487112 26324898 PMC4577153

[B9] Braunlich K. Gomez-Lavin J. Seger C. A. (2015). Frontoparietal networks involved in categorization and item working memory. Neuroimage. 107, 146–162. 10.1016/j.neuroimage.2014.11.051 25482265 PMC4306569

[B10] Brier M. R. Thomas J. B. Fagan A. M. Hassenstab J. Holtzman D. M. Benzinger T. L. . (2014). Functional connectivity and graph theory in preclinical alzheimer's disease. Neurobiol. Aging 35, 757–768. 10.1016/j.neurobiolaging.2013.10.081 24216223 PMC3880636

[B11] Calhoun V. D. Miller R. Pearlson G. Adali T. (2014). The chronnectome: time-varying connectivity networks as the next frontier in fMRI data discovery. Neuron. 84, 262–274. 10.1016/j.neuron.2014.10.015 25374354 PMC4372723

[B12] Cao H. Plichta M. M. Schafer A. Haddad L. Grimm O. Schneider M. . (2014). Test-retest reliability of fmri-based graph theoretical properties during working memory, emotion processing, and resting state. Neuroimage. 84, 888–900. 10.1016/j.neuroimage.2013.09.013 24055506

[B13] Chavez M. Valencia M. Navarro V. Latora V. Martinerie J. (2010). Functional modularity of background activities in normal and epileptic brain networks. Phys. Rev. Lett. 104, 118701. 10.1103/PhysRevLett.104.118701 20366507

[B14] Chinichian N. (2022). Investigation of Dynamical Brain Networks. PhD thesis. Berlin: Technische Universität Berlin.

[B15] Dickhoff J. (2022). Identifying Risk and Protective Factors for Suicide. University of Groningen, Groningen. 10.33612/diss.240460137

[B16] Esslinger C. Walter H. Kirsch P. Erk S. Schnell K. Arnold C. . (2009). Neural mechanisms of a genome-wide supported psychosis variant. Science. 324, 605–605. 10.1126/science.1167768 19407193

[B17] Fair D. A. Cohen A. L. Power J. D. Dosenbach N. U. Church J. A. Miezin F. M. . (2009). Functional brain networks develop from a “local to distributed” organization. PLoS Comput. Biol. 5, e1000381. 10.1371/journal.pcbi.1000381 19412534 PMC2671306

[B18] Fan L. Li H. Zhuo J. Zhang Y. Wang J. Chen L. . (2016). The human brainnetome atlas: a new brain atlas based on connectional architecture. Cerebral Cortex. 26, 3508–3526. 10.1093/cercor/bhw157 27230218 PMC4961028

[B19] Finc K. Bonna K. He X. Lydon-Staley D. M. Kühn S. Duch W. . (2020). Dynamic reconfiguration of functional brain networks during working memory training. Nat. Commun. 11, 1–15. 10.1038/s41467-020-15631-z 32415206 PMC7229188

[B20] Fornito A. Zalesky A. Bullmore E. (2016). Fundamentals of Brain Network Analysis. Cambridge, MA: Academic Press.

[B21] Fortunato S. (2010). Community detection in graphs. Phys. Rep.-Rev. Sec. Phys. Lett. 486, 75–174. 10.1016/j.physrep.2009.11.002

[B22] Fox M. D. Snyder A. Z. Vincent J. L. Corbetta M. Van Essen D. C. Raichle M. E. (2005). The human brain is intrinsically organized into dynamic, anticorrelated functional networks. Proc. Nat. Acad. Sci. 102, 9673–9678. 10.1073/pnas.0504136102 15976020 PMC1157105

[B23] Hallquist M. N. Hillary F. G. (2018). Graph theory approaches to functional network organization in brain disorders: A critique for a brave new small-world. Network Neurosci. 3, 1–26. 10.1162/netn_a_00054 30793071 PMC6326733

[B24] Harlalka V. Bapi R. S. Vinod P. Roy D. (2019). Atypical flexibility in dynamic functional connectivity quantifies the severity in autism spectrum disorder. Front. Hum. Neurosci. 13, 6. 10.3389/fnhum.2019.00006 30774589 PMC6367662

[B25] Hunter J. D. (2007). Matplotlib: A 2d graphics environment. Comput. Sci. Eng. 9, 90–95. 10.1109/MCSE.2007.55

[B26] Jeub L. Bazzi M. Jutla I. Mucha P. (2022). A generalized louvain method for community detection implemented in matlab.

[B27] Karwowski W. Vasheghani Farahani F. Lighthall N. (2019). Application of graph theory for identifying connectivity patterns in human brain networks: a systematic review. Front. Neurosci. 13, 585. 10.3389/fnins.2019.00585 31249501 PMC6582769

[B28] Lancichinetti A. Fortunato S. (2009). Community detection algorithms: a comparative analysis. Physical Rev. 80, 056117. 10.1103/PhysRevE.80.056117 20365053

[B29] Lancichinetti A. Fortunato S. (2012). Consensus clustering in complex networks. Sci. Rep. 2, 1–7. 10.1038/srep00336 22468223 PMC3313482

[B30] Leonardi N. Ville D. V. D. (2015). On spurious and real fluctuations of dynamic functional connectivity during rest. Neuroimage. 104, 430–436. 10.1016/j.neuroimage.2014.09.007 25234118

[B31] Ma Q. Tang Y. Wang F. Liao X. Jiang X. Wei S. . (2020). Transdiagnostic dysfunctions in brain modules across patients with schizophrenia, bipolar disorder, and major depressive disorder: a connectome-based study. Schizophr. Bull. 46, 699–712. 10.1093/schbul/sbz111 31755957 PMC7147584

[B32] Meunier D. Achard S. Morcom A. Bullmore E. (2009). Age-related changes in modular organization of human brain functional networks. Neuroimage. 44, 715–723. 10.1016/j.neuroimage.2008.09.062 19027073

[B33] Meunier D. Lambiotte R. Bullmore E. T. (2010). Modular and hierarchically modular organization of brain networks. Front. Neurosci. 4, 200. 10.3389/fnins.2010.00200 21151783 PMC3000003

[B34] Minamoto T. Yaoi K. Osaka M. Osaka N. (2015). The rostral prefrontal cortex underlies individual differences in working memory capacity: an approach from the hierarchical model of the cognitive control. Cortex. 71, 277–290. 10.1016/j.cortex.2015.07.025 26280275

[B35] Mucha P. J. Richardson T. Macon K. Porter M. A. Onnela J.-P. (2010). Community structure in time-dependent, multiscale, and multiplex networks. Science. 328, 876–878. 10.1126/science.1184819 20466926

[B36] Newell K. M. Mayer-Kress G. Hong S. L. Liu Y.-T. (2009). Adaptation and learning: Characteristic time scales of performance dynamics. Hum. Mov. Sci. 28, 655–687. 10.1016/j.humov.2009.07.001 19682761

[B37] Newman M. E. (2006). Modularity and community structure in networks. Proc. Nat. Acad. Sci. 103, 8577–8582. 10.1073/pnas.0601602103 16723398 PMC1482622

[B38] Owen A. M. McMillan K. M. Laird A. R. Bullmore E. (2005). N-back working memory paradigm: a meta-analysis of normative functional neuroimaging studies. Hum. Brain Mapp. 25, 46–59. 10.1002/hbm.20131 15846822 PMC6871745

[B39] Pedregosa F. Varoquaux G. Gramfort A. Michel V. Thirion B. Grisel O. . (2011). Scikit-learn: machine learning in Python. J, Mach, Learn Res. 12, 2825–2830. 10.48550/arXiv.1201.0490

[B40] Penny W. D. Friston K. J. Ashburner J. T. Kiebel S. J. Nichols T. E. (2011). Statistical Parametric Mapping: The Analysis of Functional Brain Images. Amsterdam, Netherlands: Elsevier.

[B41] Power J. D. Fair D. A. Schlaggar B. L. Petersen S. E. (2010). The development of human functional brain networks. Neuron. 67, 735–748. 10.1016/j.neuron.2010.08.017 20826306 PMC2941973

[B42] Rubinov M. Sporns O. (2010). Complex network measures of brain connectivity: uses and interpretations. Neuroimage. 52, 1059–1069. 10.1016/j.neuroimage.2009.10.003 19819337

[B43] Shirer W. R. Ryali S. Rykhlevskaia E. Menon V. Greicius M. D. (2012). Decoding subject-driven cognitive states with whole-brain connectivity patterns. Cerebral Cortex. 22, 158–165. 10.1093/cercor/bhr099 21616982 PMC3236795

[B44] Sporns O. (2010). Networks of the Brain. Cambridge, MA: MIT press. 10.7551/mitpress/8476.001.0001

[B45] Sporns O. (2012). From simple graphs to the connectome: networks in neuroimaging. Neuroimage. 62, 881–886. 10.1016/j.neuroimage.2011.08.085 21964480

[B46] Sporns O. Betzel R. F. (2016). Modular brain networks. Annu. Rev. Psychol. 67, 613–640. 10.1146/annurev-psych-122414-033634 26393868 PMC4782188

[B47] Uddin L. Q. Supekar K. S. Ryali S. Menon V. (2011). Dynamic reconfiguration of structural and functional connectivity across core neurocognitive brain networks with development. J. Neurosci. 31, 18578–18589. 10.1523/JNEUROSCI.4465-11.2011 22171056 PMC3641286

[B48] Vaiana M. Muldoon S. F. (2018). Multilayer brain networks. J. Nonlinear Sci. 1–23. 10.1007/s00332-017-9436-8

[B49] Virtanen P. Gommers R. Oliphant T. E. Haberland M. Reddy T. Cournapeau D. . (2020). SciPy 1.0: Fundamental algorithms for scientific computing in Python. Nat. Methods. 17, 261–272. 10.1038/s41592-020-0772-5 32015543 PMC7056644

[B50] Yue Q. Martin R. C. Fischer-Baum S. Ramos-Nunez A. I. Ye F. Deem M. W. (2017). Brain modularity mediates the relation between task complexity and performance. J. Cogn. Neurosci. 29, 1532–1546. 10.1162/jocn_a_01142 28471728

